# Establishment of using serum YKL-40 and SCCA in combination for the diagnosis of patients with esophageal squamous cell carcinoma

**DOI:** 10.1186/1471-2407-14-490

**Published:** 2014-07-07

**Authors:** Xin Zheng, Shan Xing, Xiao-Min Liu, Wen Liu, Dan Liu, Pei-Dong Chi, Hao Chen, Shu-Qin Dai, Qian Zhong, Mu-Sheng Zeng, Wan-Li Liu

**Affiliations:** 1 State Key Laboratory of Oncology in Southern China, Guangzhou, China; 2 Department of Clinical Laboratory, Sun Yat-sen University Cancer Center, Guangzhou, China; 3 Department of Experimental Research, Sun Yat-sen University cancer center, Guangzhou, China

**Keywords:** YKL-40, Esophageal cancer, ESCC

## Abstract

**Background:**

Elevated serum YKL-40 levels have been observed in various cancers. We evaluated the diagnostic performance of serum YKL-40 alone or in combination with the CEA, CYFRA21-1 and SCCA tumor markers for patients with esophageal squamous cell carcinoma (ESCC).

**Methods:**

YKL-40 was detected in ESCC cell lines and tissues by real-time RT-PCR, Western blotting and ELISA. YKL-40 protein expression was determined in 20 ESCC tumor tissues using immunohistochemistry. Serum YKL-40 was measured by ELISA in 126 healthy donors, 59 patients with benign esophageal diseases and 150 patients with ESCC. Serum CEA, CYFRA21-1 and SCCA were determined by electrochemiluminescence.

**Results:**

YKL-40 mRNA and protein were observed in ESCC cancer cell lines, tissues and cell culture media, respectively. YKL-40 expression was observed in 17 of 20 ESCC samples (85%). Serum YKL-40 concentration was significantly elevated in patients with ESCC (Range: 6.95-502.10 ng/ml) compared with patients with benign diseases (Range: 1.21-429.30 ng/ml; *P* = 0.038) and healthy controls (Range: 2.56-132.26 ng/ml; *P* < 0.001). ROC curves demonstrated that serum YKL-40 has a sensitivity of 72.70%, a specificity of 84.13% and an AUC of 0.874 for the diagnosis of ESCC, which was superior to CEA (Sen: 8.00%; Spe: 96.80%, AUC = 0.652), CYFRA21-1 (Sen: 40.00%; Spe: 92.06%, AUC = 0.746) and SCCA (Sen: 32.67%; Spe: 94.44%, AUC = 0.789). The YKL-40 and SCCA combination was better for diagnosing ESCC (Sen: 82.00%, Spe: 79.37%, PPV: 82.55 and NPV: 78.74; AUC = 0.917) than the YKL-40 and CEA combination (Sen: 74.00%, Spe: 83.20%, PPV: 84.09 and NPV: 72.73; AUC = 0.877), the YKL-40 and CYFRA21-1 combination (Sen: 82.00%, Spe: 77.78%, PPV: 81.46% and NPV: 78.40%; AUC = 0.897) or the CEA, CYFRA21-1 and SCCA combination (Sen: 56.67%, Spe: 84.80%, PPV: 81.73 and NPV: 61.99; AUC = 0.831). Associations between serum YKL-40 levels and the clinic characteristics of ESCC were not significant, with the exception of age (*p* = 0.001).

**Conclusions:**

ESCC tumor cells and tissues express YKL-40. Serum YKL-40 may be a potential biomarker for ESCC. Serum YKL-40 in combination with SCCA significantly increases the sensitivity of detecting ESCC.

## Background

Esophageal squamous cell carcinoma (ESCC) is typically diagnosed at a late state and therefore has a very high mortality rate. It is the sixth leading cause of cancer mortalities worldwide [1]. The overall 5-year survival rate for patients treated with surgery alone is less than 20%, with a median survival of 13 to 17 months [2-5]. Currently, traditional tumor markers, such as CEA, CYFRA21-1 and SCCA, are used to diagnose and evaluate ESCC progression. However, these tumor markers exhibit a low sensitivity in detecting ESCC. Kawaguchi H demonstrated that the diagnostic sensitivity of CEA was only 17.0% in ESCC [6]. Mealy K reported that the individual sensitivities of CEA and SCCA for the diagnosis of ESCC were about 28% and 32%, respectively [7]. Yamamoto K study demonstrated that the sensitivity of CYFRA 21-1 was only 47.9%, although the specificity was 100% [8]. Likewisely, our previous study reported that CEA and CYFRA21-1 exhibited sensitivities of 13.4% and 32.1% for the detection of ESCC, respectively [9]. These results indicate that the sensitivity of the traditional ESCC tumor markers is too low to diagnose esophageal cancer diagnosis. Therefore, there is an urgent need to identify tumor markers to improve the sensitivity of ESCC detection.

YKL-40, a secreted glycoprotein, belongs to a group of mammalian proteins with an amino acid sequence that is similar to the 18-glycosyl hydrolase group of bacterial chitinases [10]. It is secreted by various human cells, such as synovial, cartilage, endothelial, neutrophil and macrophage cells [11]. YKL-40 is involved in angiogenesis, growth, proliferation, differentiation, and remodeling processes [12]. Serum YKL-40 levels are elevated in pathological conditions, including inflammation and cancer [13,14]. Recently, YKL-40 was reported to be highly expressed in several types of cancers, including ovarian cancer [15], breast cancer [16], lung cancer [17], hepatocellular carcinoma [18], and glioblastoma [19]. In addition, serum YKL-40 has been suggested as a potential biomarker for the diagnosis and monitoring of these cancers [20-24].

The diagnostic value of serum YKL-40 in patients with ESCC remains unknown. The goal of our present study is to investigate the levels of YKL-40 expression in ESCC tumor cells and to evaluate the diagnostic performance of serum YKL-40 in ESCC diagnosis compared with the traditional ESCC tumor markers CEA, CYFRA21-1 and SCCA.

## Methods

### Cell lines

The immortalized esophageal epithelial cell line NE-3, induced by human papillomavirus type 16 E6/E7, was obtained from Dr. Jin (the University of Hong Kong, P.R. China) and was cultured in Keratinocyte-SFM (Invitrogen, Carlsbad, CA) media [25,26]. The ESCC cell lines Eca-109, Kyse30, Kyse140, Kyse180, Kyse510 and Kyse520 (Chinese Academy of Sciences, Shanghai, China) were grown in RPMI 1640 (Invitrogen, USA) supplemented with 10% fetal bovine serum [26].

### Serum and tissue specimen

Serum from 150 ESCC patients (ages 30-96 years, median 58 years) was collected at the time of diagnosis before tumor resection at the Cancer Center of Sun Yat-Sen University from 2002 to 2005. The patient characteristics are described in Table 1. The absence of disease such as COPD and second primary carcinomas was assessed by clinical history, physical examination, routine laboratory tests (including liver and renal function tests), and colonoscopy. Serum from 126 healthy donors without inflammation (ages 22-78 years, median = 54 years, 74 males and 52 females) were collected from the physical examination department at the Cancer Center of Sun Yat-Sen University. Serum of 59 patients (ages 21-80 years, median = 55 years, 35 males and 24 females) with benign esophageal disease (40 cases of reflux esophagitis, 6 cases of acute suppurative esophagitis and 13 cases of esophageal hiatal hernia) were collected at the first affiliated hospital of Sun Yat-sen University. Venous blood (3-5 ml) was obtained at the time of diagnosis before treatment, clotted at room temperature, centrifuged at 3000 r/min for 10 min and stored at -80°C until use.

**Table 1:** Levels of YKL-40 and clinical characteristics of patients with ESCC

**Characteristics**	**Case numbers**	**YKL-40(ng/ml)**	
		**Median(range)**	**p Value**^**a**^
Age, years			
<60	81	71.56(6.95-340.70)	0.001
≥60	69	122.36(21.32-502.05)	0.001
Gender			
Male	113	93.12(11.56-430.83)	0.784
Female	37	111.60(6.95-502.05)	0.784
pT status			
pT1	5	93.12(21.32-264.66)	0.975
pT2	21	104.27(30.26-430.83)	0.975
pT3	65	91.55(6.95-421.34)	0.975
pT4	45	101.50(14.64-351.66)	0.975
pN status			
pN0	57	97.27(6.95-430.83)	0.617
pN1	77	97.03(13.32-421.34)	0.617
pM status			
pM0	107	93.24(6.95-430.83)	0.198
pM1	32	108.82(14.64-419.22)	0.198
pTNM status			
Stage I	7	93.12(42.26-264.66)	0.604
Stage II	40	97.27(6.95-430.83)	0.604
Stage III	58	92.40(13.32-421.34)	0.604
Stage IV	32	108.82(14.64-419.22)	0.604
Tumor grade			
Grade 1	24	108.39(24.95-234.89)	0.579
Grade 2	57	94.21(11.56-376.09)	0.579
Grade 3	41	71.02(6.95-419.22)	0.579

^a^Kruskal-Wallis test.

A total of 20 formalin-fixed and paraffin-embedded ESCC tumor specimens for immunochemistry were obtained at the Sun Yat-sen University Cancer Center from November of 2012 to December of 2013. Six pairs Real-time RT-PCR and Western-blotting tissue samples were obtained from 2011 to 2013. The corresponding normal esophageal tissue specimens (n = 20) were taken from areas a standard distance (8 cm) from the corresponding resected tumors. All these ESCC and carcinoma-adjacent tissue samples were collected immediately after surgical resection and confirmed by pathological review.

Prior to use of these serum and tissues, informed consent was obtained from each of the participants. All patients provided written informed consent. This experiment was approved by the Institute Research Ethics Committee of the Cancer Center of Sun Yat-Sen University, Guangzhou, China.

### Real-time RT-PCR

Total RNA was extracted from cell lines and frozen ESCC tissues using the Trizol reagent (Invitrogen, USA) according to the manufacture’s instruction.

Reverse transcription of total RNA (2 μg) was done using SuperScript II reverse transcriptase. The quantification of target and reference (GAPDH) genes was performed in triplicate on a LightCycler® 480 II (Roche, Applied Science) using a SYBR green-based assay (BioRad, USA). The primers used in the real-time RT-PCR reaction were as follows: YKL-40 forward 5′- GAGGATGGAACTTTGGGTCTC-3′ and reverse 5′- TCATTTCCTTGATTAGGGTGGT-3′; and GAPDH, forward 5′-GACTCATGACCACAGTCCATGC-3′ and reverse 5′-AGAGGCAGGGATGATGTTCTG-3′.

### Western blotting analysis

Western blot analysis was performed via standard protocols with antibodies to YKL-40 and α-tublin (Abcam, UK).

### Immunohistochemistry

Formalin-fixed, paraffin-embedded ESCC sections were incubated with a rabbit polyclonal anti-YKL-40 antibody (1:100, Bioss, China) overnight at 4°C. After washing in PBST, the tissue sections were treated with a horseradish peroxidase-conjugated anti-rabbit secondary antibody (1:1000, Zymed). The tissue sections were then developed with 3-diaminobenzidine tetrahydrochloride for 10 seconds, followed by counterstaining with 10% Mayer’s hematoxylin. The degree of immunostaining was reviewed by two independent observers.

### ELISA

Serum YKL-40 levels were determined by double-antibody sandwich ELISA according to the manufacturer’s instructions (R&D systems, USA). Briefly, 96-well microplates were coated with 100 μl/well of the capture antibody (rat anti-human YKL-40, 2.0 μg/ml) overnight at 4 C. After blocking with 3% BSA, 100 μl of the test samples (1:100 diluted in 1% BSA) was added and incubated for 2 h at room temperature. Subsequently, 100 μl/well of the detection antibody (biotinylated goat anti-human YKL-40, 200 ng/ml) was added and incubated for 2 h at room temperature. Next, 100 μl/well of Streptavidin-HRP (1:200) was added and incubated for 20 min at room temperature. Finally, the substrate (tetramethylbenzidine) solution was added, and the reaction was stopped with 2 N H_2_SO_4_ and read at an OD of 450 nm. Each test included a standard control (CV = 12%).

### CEA, CYFRA21-1 and SCCA assay

The concentrations of CEA and CYFRA21-1 in the serum were assessed using electrochemiluminescence immunoassay (ECLIA) kits (CEA, lot: 172356; CYFRA21-1, lot: 169393; Roche, German) on a Roche E170 fully automatic electrochemistry luminescence immunity analyzer (Roche, German). The levels of SCCA in the serum were detected using an ARCHITECT I2000SR immune analyze system (Abbott, America) (SCCA, lot: 34111LP68; Abbott, America). Each test included a standard control (CV < 5%).

### Statistical analysis

Statistical analyses were performed with the SPSS 16.0 (SPSS Inc.) The relationships between the expression of YKL-40 protein and the clinicopathologic features were analyzed by the Mann-Whitney U test. The comparisons of YKL-40 concentration among different groups were assessed using the Kruskal-Wallis test. The efficacy of YKL-40 was evaluated by the area under receiver operating characteristic (ROC) curve (AUC). The cut-off value for YKL-40 was defined as the value with the maximization of the Yuden index. Furthermore, sensitivity (Sen), specificity (Spe), positive predictive value (PPV) and negative predictive value (NPV) were used to compare the efficiency of diagnosis among YKL-40, CEA, CYFRA21-1 and SCCA. All statistical tests were two-sided, and p < 0.05 was considered statistically significant.

## Results

### Expression of YKL-40 in esophageal carcinoma cell lines and tumor tissues

**Figure 1:**
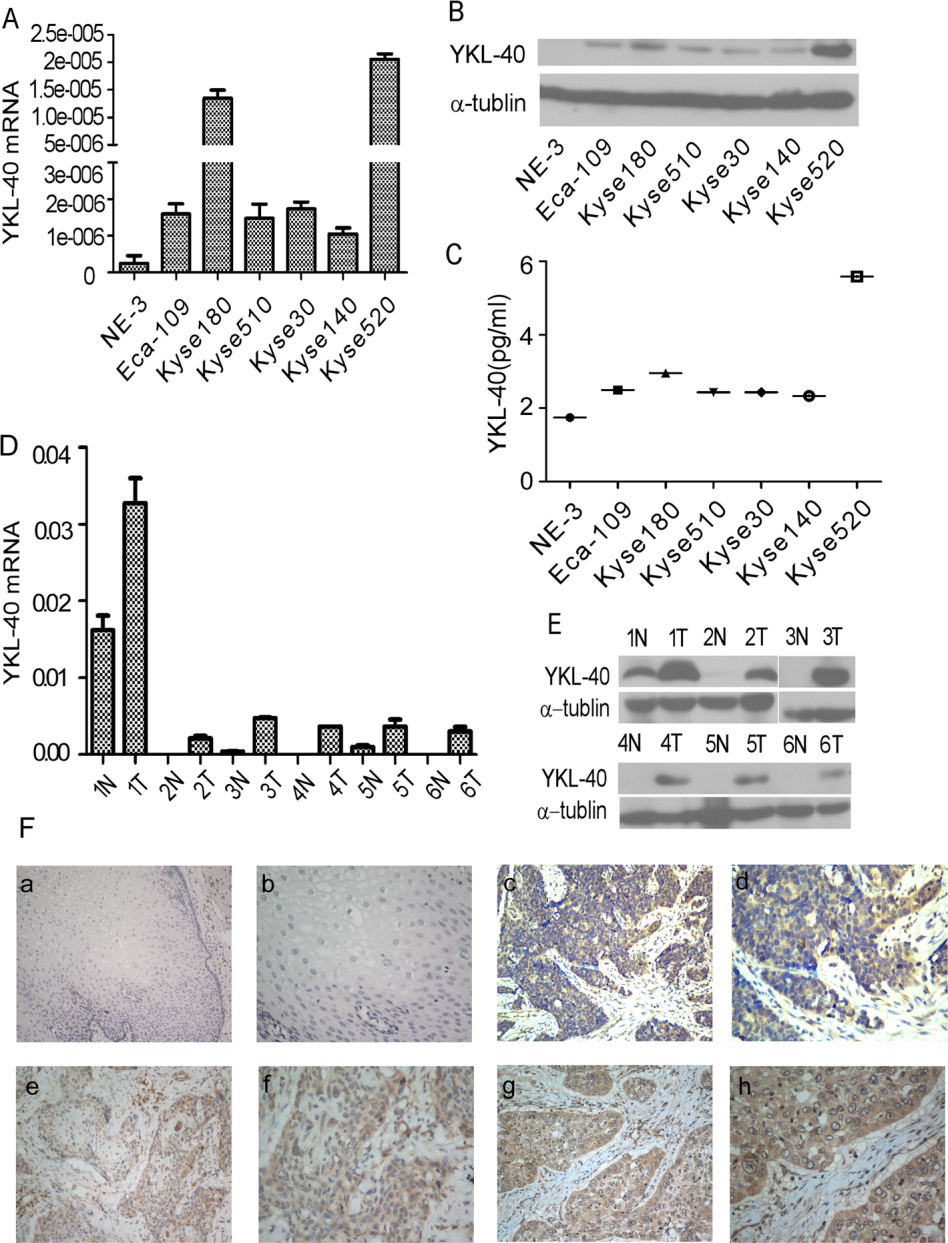
**Expression of YKL-40 mRNA or protein in ESCC cell lines, tissues and location in tissue.** Expression of mRNA and protein in immortalized esophageal epithelial cell line (NE-3) and esophageal carcinoma cell lines was analyzed by real-time PCR and Western Blotting, respectively **(A, B)** and in six pairs of matched ESCC and noncancerous tissues **(D, E)**. Expression level was normalized by GAPDH and α-tublin, respectively. Error bars represent standard deviations (SD) calculated from three parallel experiments. Protein level in supernatant was measured by ELISA **(C)**. Location of YKL-40 was determined by immunohistochemistry **(F)**. The normal esophageal epithelial tissue showed no expression of YKL-40 (**F a-b**, 200 × and 400×). The ESCC tissues showed low **(F c-d)**, medium **(F e-f)** and high **(F g-h)** expression of YKL-40 (200× and 400×).

To investigate the expression of YKL-40 in ESCC, the YKL-40 mRNA and protein levels were detected by real-time RT-PCR and Western Blotting, respectively, in several esophageal carcinoma cell lines (Eca-109, Kyse180, Kyse510, Kyse30, Kyse140 and Kyse520) and the immortalized esophageal epithelial cell line NE-3. As shown in Figure 1A and B, higher YKL-40 mRNA and protein levels were observed in all tumor cell lines compared with the immortalized cell line NE-3. Next, we performed a double-antibody sandwich ELISA to determine the protein expression of YKL-40 in the media of the cell lines. The levels of YKL-40 protein observed in the media of the esophageal cancer cell lines were relatively low level but were still higher than that of NE-3 (Figure 1C). Thus, consistent with the results of the mRNA analysis, YKL-40 protein expression was up-regulated in the ESCC cell lines. Furthermore, comparative analysis of YKL-40 expression was conducted on six pairs of matched ESCC tissue and adjacent noncancerous tissue. The expression of YKL-40 mRNA in the six ESCC samples was much higher than the paired adjacent noncancerous tissue (Figure 1D). Similarly, the expression level of YKL-40 protein was also increased in ESCCs compared with that in the adjacent nonmalignant esophageal tissues (Figure 1E).To further investigate the exact expression state of YKL-40 in vivo, YKL-40 protein expression was determined by immunohistochemistry. YKL-40 protein was detected in 17 of 20 ESCC samples (85%) but not in the normal esophageal epithelium (Figure 1F). YKL-40 was mainly located in the cytoplasm of tumor cells, as well as in the tumor stroma cells. The expression levels of YKL-40 in tumor cells was observed at various levels: low (Figure 1F c-d), medium (Figure 1F e-f) and high (Figure 1F g-h). The high expression of YKL-40 was also observed in mesenchymal cells surrounding carcinoma (Figure 1F g-h).

### Serum YKL-40 levels in ESCC and the association between serum YKL-40 and clinicopathological characteristics

**Figure 2:**
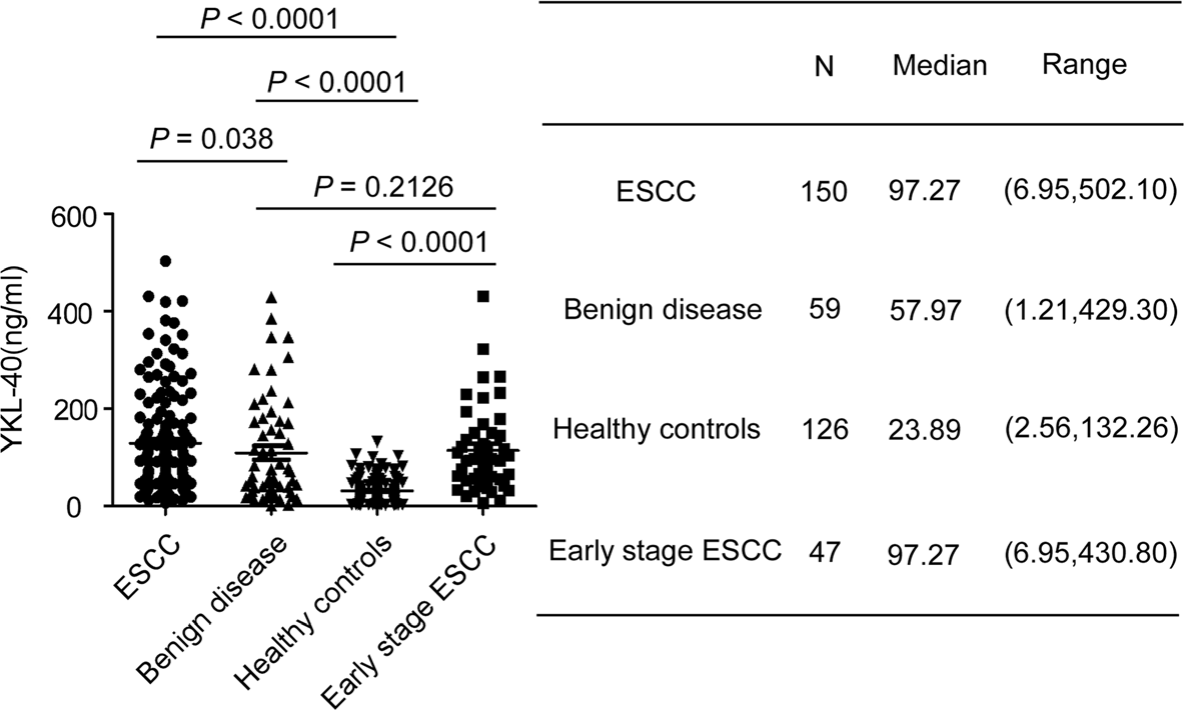
**YKL-40 concentration in serum in the test cohort.** Left side, the serum YKL-40 in ESCC patients, benign disease patients, healthy controls and early-stage ESCC patients are plotted as a distribution. P value was calculated using Kruskal-Wallis test. Right side, YKL-40 serum levels in different groups.

Figure 2 presents the serum levels of YKL-40 in the patients with ESCC (n = 150), patients with benign esophageal diseases (n = 59), healthy controls (n = 126) and patients with early-stage ESCC (n = 47, 7 cases of stage I, 40 cases of stage II). The mean YKL-40 level was 97.27 ng/ml (range, 6.95-502.10) in patients with ESCC, 57.97 ng/ml (range, 1.21-429.30) in patients with benign esophageal diseases, 23.89 ng/ml (range, 2.56-132.26) in healthy controls and 97.27 ng/ml (range, 6.95-430.80) in early stage ESCC. The serum levels of YKL-40 in patients with ESCC were significantly higher than those of healthy control subjects (*p* < 0.001) and those of patients with benign disease (*p* = 0.038), and the serum levels of YKL-40 of the early-stage ESCC patients were significantly higher than healthy control subjects (*p* < 0.001) but similar to benign disease patients (*p* = 0.2126) (Figure 2).

The associations between the median serum YKL-40 levels and the clinicopathological parameters are presented in Table 1. Serum YKL-40 was not significantly correlated with gender, T classification, N classification, metastasis, clinical stage or tumor grade. However, there was a significant association between the level of serum YKL-40 and age (*p* = 0.001). The level of serum YKL-40 was higher in elder patients (≥60) than in patients below the age of 60.

### Diagnostic values of individual serum YKL-40, CEA, CYFRA21-1 and SCCA levels or combinations in the detection of ESCC

**Figure 3:**
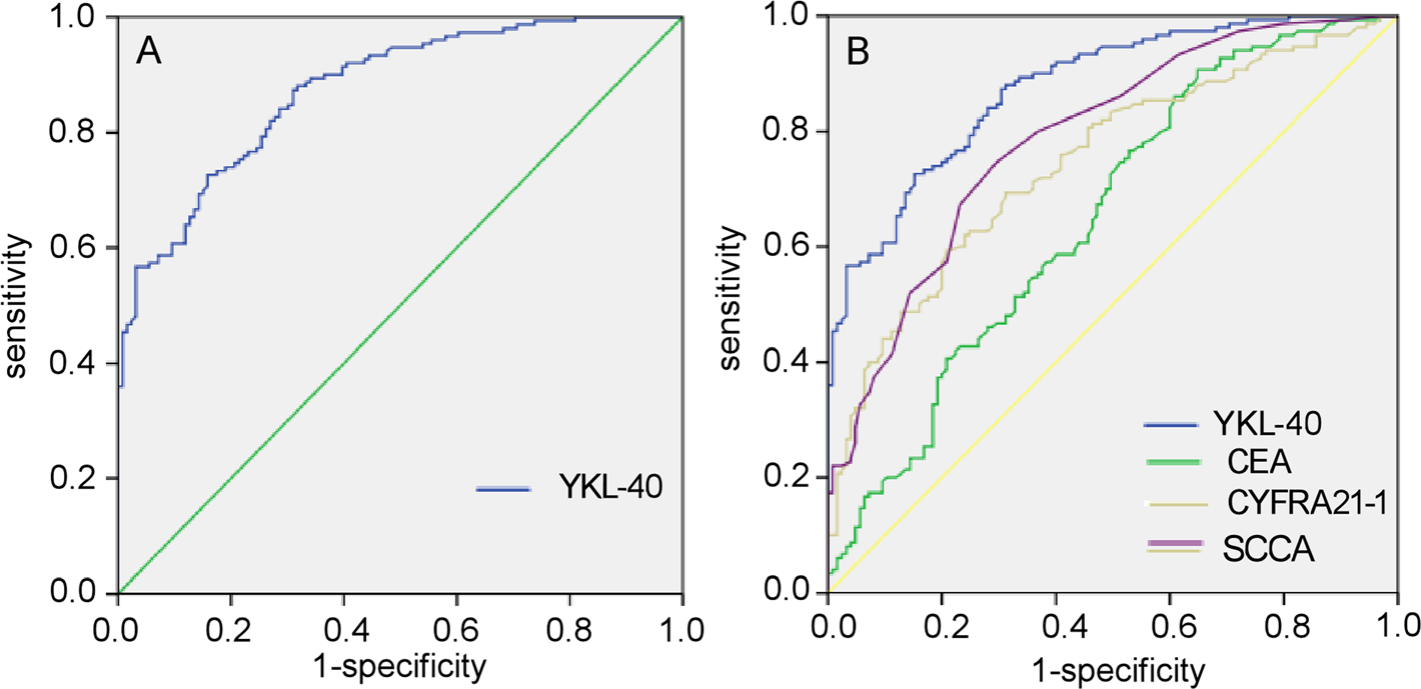
**Diagnostic outcomes for serum YKL-40, CEA, CYFRA21-1 or SCCA in the diagnosis of ESCC. A**. ROC curves of the serum YKL-40 levels of 150 ESCC patients and 126 controls. The estimated area under the ROC curve was observed as AUC = 0.874. **B**. ROC curves for the diagnostic strength to identify ESCC using YKL-40, CEA, CYFRA21-1 or SCCA (CEA: AUC = 0.652; CYFRA21-1 = 0.746; SCCA = 0.789).

The ROC curve was plotted to identify a cut-off value that could distinguish 150 ESCC patients from 126 healthy controls. As shown in Figure 3, the AUC of YKL-40 was 0.874 (95% CI: 0.792–0.885), with an optimal cut-off value 58.0 ng/ml, whereas the AUCs of CEA, CYFRA21-1 and SCCA were 0.652 (95% CI: 0.593–0.708), 0.746 (95% CI: 0.691–0.797) and 0.789(95% CI: 0.736–0.842), respectively. The cut-off values that we applied for CEA, CYFRA21-1 and SCCA were 5.0 ng/ml, 3.3 ng/ml and 1.5 ng/ml, respectively, according to the manufacturer’s protocols. As shown in Table 2, the sensitivity of YKL-40 was 72.70%, which is significantly higher than that for CEA (8.00%), CYFRA21-1 (40.00%) and SCCA (32.67%), whereas the specificity of serum YKL-40 was slightly lower. Moreover, serum YKL-40 exhibited a higher NPV compared with CEA, CYFRA21-1 and SCCA (72.11% vs. 46.72% vs. 56.31% vs. 54.09%) without an obvious reduction in the PPV.

**Table 2:** Diagnostic values, including sensitivity, specificity, positive predictive value and negative predictive value, combining assay seromarkers

**Combinations**	**Sensitivity(%)**	**Specificity(%)**	**PPV(%)**	**NPV(%)**
YKL-40	72.70	84.13	84.50	72.11
CEA	8.00	96.80	75.00	46.72
CYFRA21-1	40.00	92.06	85.71	56.31
YKL-40 or CEA	74.00	83.20	84.09	72.73
YKL-40 or CYFRA21-1	82.00	77.78	81.46	78.40
CEA or CYFRA21-1	44.00	89.60	69.47	57.14
YKL-40 or CEA or CYFRA21-1	83.33	76.80	81.17	79.34

Cut-off values: 58.0 ng/ml for YKL-40; 5.0 ng/ml for CEA; 3.3 ng/ml for CYFRA21-1; 1.5 ng/ml for SCCA.

**Figure 4:**
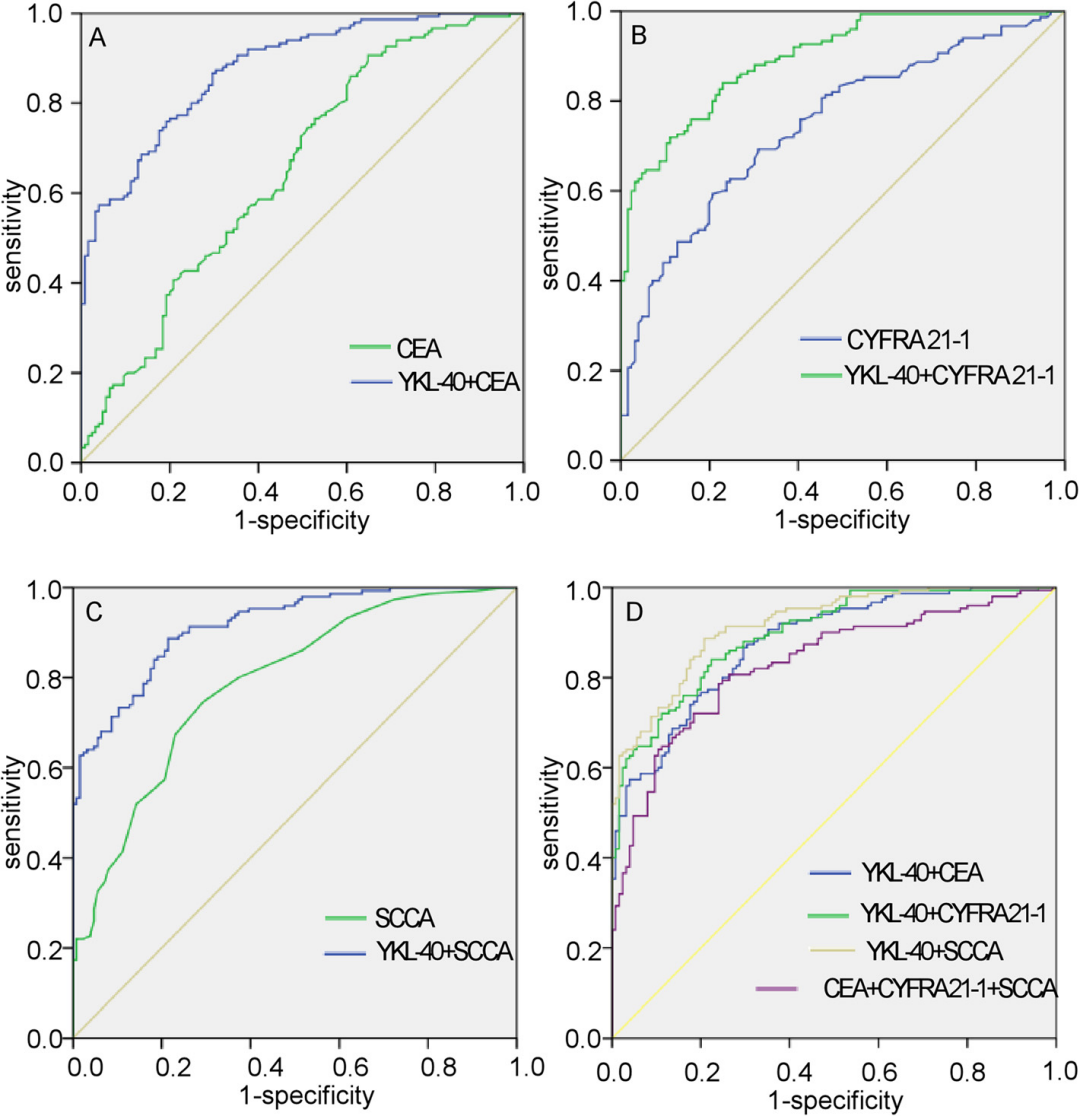
**Diagnostic outcomes for serum YKL-40, CEA, CYFRA21-1 or SCCA combination in the diagnosis of ESCC. A**. ROC curves for the diagnostic strength to identify ESCC using CEA and YKL-40+CEA (CEA: AUC=0.652; YKL-40+CEA: AUC=0.877). **B**. ROC curves for the diagnostic strength to identify ESCC using CYFRA21-1 and YKL-40+CYFRA21-1 (CYFRA21-1: AUC=0.746; YKL-40+CYFRA21-1: AUC=0.897). **C**. ROC curves for the diagnostic strength to identify ESCC using SCCA and YKL-40+SCCA (SCCA: AUC=0.789; YKL-40+SCCA: AUC=0.917). **D**. ROC curves for the diagnostic strength to identify ESCC using YKL-40+CEA, YKL-40+CYFRA21-1, YKL-40+SCCA and CEA+CYFRA21-1+SCCA (YKL-40+CEA: AUC=0.877; YKL-40+CYFRA21-1: AUC=0.897; YKL-40+SCCA: AUC=0.917; CEA+CYFRA21-1+SCCA: AUC=0.831).

To further improve diagnostic accuracy, we used parallel combinations to establish models with the above-mentioned seromarkers. That is, the sample would be defined as positive for ESCC if any of the markers in the combination was above the cut-off value. Table 2 demonstrates that the sensitivity of the combination of YKL-40 and SCCA (82.00%) was superior to that of the combination of CEA, CYFRA21-1 and SCCA (56.67%) or that of YKL-40 and CEA (74.00%) but was similar to that of the YKL-40 and CYFRA21-1 combination (82.00%). The specificity of the combination of YKL-40 and SCCA (79.37%) was slightly lower than the combination of CEA, CYFRA21-1 and SCCA (84.80%) or the combination CEA and YKL-40 (83.20%) and slightly higher than that of the YKL-40 and CYFRA21-1 combination (77.78%). The combination of YKL-40 and SCCA (78.74%) exhibited a better NPV than that of the combination of CEA, CYFRA21-1 and SCCA (61.99%) or the combination of CEA and YKL-40 (72.73%) or the combination of YKL-40 and CYFRA21-1 (78.40%). The PPV of the combination of CEA, CYFRA21-1 and SCCA (81.73%) was similar to that of the combination of YKL-40 and CYFRA21-1 (81.46%) or the combination of YKL-40 and SCCA (82.55%) and was slightly lower than the combination of CEA and YKL-40 (84.09%). ROC analysis also demonstrated that the addition of YKL-40 to either tumor marker significantly increased the AUC of detection of ESCC (YKL-40 + CEA vs. CEA = 0.877 vs. 0.652; YKL-40 + CYFRA21-1 vs. CYFRA21-1 = 0.897 vs. 0.746; YKL-40 + SCCA vs. SCCA = 0.917 vs. 0.789). Moreover, YKL-40 in combination with SCCA had the highest classification accuracy among the models with the seromarkers (CEA + CYFRA21-1 + SCCA: AUC = 0.831; YKL-40 + CEA: AUC = 0.877 YKL-40 + CYFRA21-1: AUC = 0.897; YKL-40 + SCCA: AUC = 0.917) (Figure 4).

Similarly, Table 3 demonstrates that YKL-40 improved the sensitivity (70.21%) significantly in detecting early-stage ESCC compared with the individual tumor markers (CEA: 10.64%; CYFRA21-1: 40.43%; SCCA: 29.79%), and the combination of YKL-40 and SCCA was superior to the other combinations in the efficiency of diagnosing ESCC.

**Table 3:** Diagnostic values in early stage ESCC combining assay seromarkers

**Combinations**	**Sensitivity(%)**	**Specificity(%)**	**PPV(%)**	**NPV(%)**
YKL-40	70.21	84.13	62.26	88.33
CEA	10.64	96.80	55.56	74.23
CYFRA21-1	40.43	92.06	65.52	80.56
YKL-40 or CEA	70.21	83.20	61.11	88.14
YKL-40 or CYFRA21-1	78.72	77.78	56.92	90.74
CEA or CYFRA21-1	46.80	89.60	62.86	81.75
YKL-40 or CEA or CYFRA21-1	78.72	76.80	56.06	90.57

Cut-off values: 58.0 ng/ml for YKL-40; 5.0 ng/ml for CEA; 3.3 ng/ml for CYFRA21-1; 1.5 ng/ml for SCCA.

## Discussion

In the present study, we found that YKL-40 protein is expressed in ESCC cell lines and ESCC tumor tissues. Serum YKL-40 levels were significantly elevated in patients with ESCC compared with patients with benign diseases and healthy controls. Serum YKL-40 in combination with SCCA significantly increased the sensitivity of detecting ESCC compared with the traditional ESCC tumor markers CEA, CYFRA21-1 and SCCA.

A number of studies have reported that YKL-40 is expressed in tumor cells [27-30]. Due to post-transcriptional regulation, there are some inconsistencies between mRNA and protein expression among the esophageal cancer cell lines. However, in general, YKL-40 was up-regulated in esophageal cancer cell lines and tumor tissue both at the transcriptional and translational level compared to the immortalized esophageal epithelial cell line NE-3 and paired adjacent noncancerous tissue, respectively. Subsequently, using immunohistochemistry analysis, YKL-40 expression was observed in 17 (85.0%) of 20 ESCC tumor tissues but not in neighboring normal esophageal epithelium. These data suggested that YKL-40, expressed in ESCC tumor cells and secreted into the media of tumor cell culture, may be a candidate tumor marker for the detection of ESCC.

Because YKL-40 is a secreted protein expressed in tumor cells, it has been investigated as a tumor marker in many types of cancers. In this study, we tested whether serum YKL-40 could be used as a tumor marker for ESCC. Serum YKL-40 levels in the ESCC group were much higher than in healthy controls. Considering that elevated serum YKL-40 levels were observed in patients with inflammation and the possible influence of chronic inflammation, we examined YKL-40 expression in a set of patients with benign esophageal disease and accompanying chronic inflammation (N = 59) to study whether inflammation would affect the serum levels of YKL-40. Our results demonstrated that the serum YKL-40 levels of patients with benign diseases were significantly higher than those of healthy controls (*p* < 0.0001) but significantly lower than those of the ESCC group (*p* = 0.038). These data indicate that patients with benign disease and elevated serum YKL-40 levels exhibit inflammation and that ESCC patients express higher levels of serum YKL-40 than do patients with benign diseases. A number of studies have demonstrated that the development of ESCC is associated with chronic inflammation [31-34]. Our previous study and others determined that the inflammation markers SAA and CRP are significantly elevated in patients with ESCC [9]. Both ESCC tumor cells secreting YKL-40 and inflammation factors increasing YKL-40 expression may account for the higher serum levels of YKL-40 observed in ESCC patients. Our data show that YKL-40 is not able to distinguish between patients with benign disease and early-stage ESCC (p = 0.2126), possibly due to our small sample size of early-stage ESCC patients, which was caused by the difficultly in achieving early diagnosis. In addition, we observed no correlation between the preoperative serum level of YKL-40 and patient disease characteristics, with the exception of age (*p* = 0.001). There was no significant difference in serum YKL-40 levels between patients with early-stage tumors (I-II) and patients with advanced-stage tumors (III-IV). These results indicate that serum YKL-40 can be used for the detection early ESCC as well as for the detection advanced ESCC.

CEA, CYFRA21-1 and SCCA are the most commonly investigated tumor markers for the diagnosis of ESCC [7]. In this study, ROC curve analysis revealed that the accuracy of serum YKL-40 for the diagnosis of ESCC was superior that of CEA, CYFRA21-1, and SCCA. In line with previous studies [6-9], CEA, CYFRA 21-1 and SCCA exhibited low sensitivity but high specificity for ESCC detection in our study. However, compared with CEA, CYFRA 21-1 and SCCA, serum YKL-40 exhibited higher sensitivity and slightly lower specificity. The effect of inflammation factors on the serum levels of YKL-40 may have led to the lower specificity of serum YKL-40 in the diagnosis of ESCC.

CEA, CYFRA 21-1 and SCCA alone exhibit low sensitivity for the diagnosis of ESCC. Researchers have demonstrated that combinations of tumor markers can marginally improve diagnostic efficacy compared with single markers [7,35,36]. In the present study, the addition of YKL-40 to CEA (74.00%), CYFRA21-1(82.00%) or SCCA (82.00%) increased the diagnostic sensitivity compared with CEA (8.00%), CYFRA21-1 (40.00%) or SCCA (32.67%) alone, but the diagnostic specificity did not significantly decrease. Consistent with the report of Munck-Wikland et al. [37], our results demonstrated that reliance on the three traditional tumor markers CEA, CYFRA21-1 and SCCA for the detection of ESCC is not satisfactory, especially in light of the poor sensitivity (46.81%). However, the combination of YKL-40 and SCCA significantly improved the sensitivity of the detection of ESCC and was superior to the sensitivity of the three traditional tumor markers CEA, CYFRA21-1 and SCCA. Moreover, the YKL-40 and SCCA combination increased the NPV, which can more accurately differentiate patients from healthy individuals. ROC analysis also confirmed that YKL-40 in combination with SCCA was the best model for discriminating between ESCC cases and controls. Moreover, YKL-40 combined with SCCA also served as a more sensitive tumor maker for the detection of patients with early-stage ESCC. Although an analysis of additional patients is needed to verify and expand the present results, our data indicate that the addition of YKL-40 to the traditional ESCC tumor marker SCCA may significantly improve the sensitivity of the detection of ESCC.

## Conclusions

In conclusion, our research indicated that YKL-40 is up-regulated in ESCC tumor and that patients with ESCC exhibit elevated levels of serum YKL-40. YKL-40 in combination with SCCA significantly improves the sensitivity of traditional the ESCC tumor markers CEA, CYFRA21-1 and SCCA in the detection of ESCC.

## Abbreviations

YKL-40: Chitinase-3-like-1 protein; SCCA: Squamous cell carcinoma antigen; CYFRA21-1: Cytokeratin 19 fragments; CEA: Carcino-embryonic antigen; ESCC: Esophageal squamous cell carcinoma; COPD: Chronic obstructive pulmonary diseases.

## Competing interests

The authors declare that they have no competing interests. There are no non-financial competing interests (political, personal, religious, ideological, academic, intellectual, commercial or any other) to declare in relation to this manuscript.

## Authors’ contributions

In these studies, XZ and SX carried out the main work and contributed equally. They participated in the design of the study and performed the statistical analysis and drafted the manuscript. XML carried out the immunoassays. WLL and other authors conceived of the study, and participated in its design and coordination and helped to draft the manuscript. All authors read and approved the final manuscript.

## Pre-publication history

The pre-publication history for this paper can be accessed here:

http://www.biomedcentral.com/1471-2407/14/490/prepub
